# Continuation of the “Case of Facial Palsy Consequent upon Otitis,” Reported for the December Number, 1849, of This Journal

**Published:** 1851-01

**Authors:** W. H. Cobb

**Affiliations:** Louisville, Ky.


Art. III.—*Continuation of the "Case of Facial Palsy consequent upon Otitis,"* reported for the December number, 1849, of this Journal. By W. H. Cobb, M. D., of Louisville, Ky.

By reference to the title of this article, it will be perceived that it is a supplement to a case of Facial Palsy published in December, 1849.

Lest any of my readers may not have seen the case referred to, or the circumstances have escaped the recollection of those who did read it, I have deemed it proper to recapitulate, in a brief and cursory manner, some of the more important symptoms.

The patient, Thomas Allen, 31 years of age, a man of small stature, and infirm health, entered the Louisville Marine Hospital in the month of September, 1849, for the relief of irritability of the bladder.

Oct. 7th. While the patient was still within the Institution, an acute attack of inflammation was ingrafted upon a chronic affection, of 5 months standing, of the left ear. A discharge of purulent matter from the left ear had existed at the time of his entrance, but as it seemed to give him but little uneasiness, no special treatment was directed towards it.

Oct. 20th. The paralysis of the facial muscles, combined with a slight degree of anæsthesia was noticed today for the first time. In reviewing the symptoms of the case, I ventured to predict that the palsy was probably owing to one of two causes—1st, ulceration may have extended to the substance of the Portio Dura while lodged in the Aqueducts Fallopii of the Temporal bone—or, 2nd, that the palsy was owing to compression of the nervous filamento by a piece of necrosed bone, or an abscess. At the time of making the last report (Nov. 10th) I had no idea that an opportunity would so soon be afforded me of verifying the conjectures as to the probable cause; the patient was gradually though slowly improving in his general health, his appetite had returned, and his strength was so much invigorated that he was enabled to walk about the wards, and even to perform the lighter duties required of him by the nurse. Still the motion of the palsied muscles did not return, the anesthesia continued, and the discharge from the left ear, though somewhat diminished in quantity, never entirely ceased.

The treatment adopted, was the internal exhibition of chalybeate tonics, counter irritation, and the endermic application of Strychnine over the pes anserinus, with exercise in the open air whenever the state of the weather would permit. About this period, he was unintentionally salivated by a purgative of calomel and rhubarb, notwithstanding the prompt exhibition of a dose of Ol. Ricini, when it was found that the medicine would not be likely to operate; the salivation however seemed to operate beneficially upon the disease, as the motion of the palsied muscles was to a slight extent restored. Still the patient, notwithstanding the manifest improvement in his strength, appetite, &c., suffered from frequent attacks of acute supra orbital pain of such violence as (using his own expression) to almost drive him distracted. Chloroform applied externally was the only remedy that seemed to have any effect in allaying them.

Dec. 1st.—7, P. M. The patient complained of intense pain in the head, but as this was no unusual circumstance, no particular attention was paid to him. His pulse was slow (the number of beats was not counted,) and rather weak, pupils slightly dilated, skin of the trunk and face warmer than natural, extremities cool, bowels open. The discharge from the ear affected, had diminished somewhat in quantity, as well as changed in quality, becoming thinner and more serous in its character. His mind was perfectly sane, as he answered questions rationally and coherently. A compress, wet in cold water, was applied to the scalp and warm applications made to the feet.

Dec. 2nd. The patient, according to the report of the nurse, had an attack of shivering, shortly after my last visit, of so marked a character as to attract the attention of the patients occupying the adjoining beds. He was constantly putting his hands to his head, rolling from side to side, and endeavoring to get out of bed. Delirium, of a low, muttering character, supervened about 11 o'clock at night.

*Actual condition at time of visit.*—Still delirious, and constantly muttering the words "Bonny Canny," hands frequently applied to forehead, countenance expressive of great agony, pupils slightly contracted, skin of face dry and warm, tongue clean and moist, pulse 60, weak; bowels open, urine freely voided, extremities cold. Ordered, by Professor Gross, milk punch—a large blister to be applied to the nape of the neck, and the extremities to be immersed in a mustard bath, and afterwards to be kept warm, by jugs of hot water.

Dec. 2nd.—7, P. M. Patient still delirious, picking and pulling the bed-clothes, eyes affected with strabismus, pupils dilated and insensible to the action of light, surface of the whole body bedewed with a cold clammy perspiration, pulse, slightly increased in frequency, feeble.

Dec. 3d.—9, A. M. Patient lies constantly upon the left side; when laid upon his right side, he immediately resumes his former position. Symptoms same as last night, though in a more aggravated form. Pulse imperceptible at the wrist, two involuntary evacuations during the night. He continued in this state till about 3 o'clock, P. M., of the same day, when he died.

*Postmortem.* Appearances.—Body much emaciated. Face swollen and distorted. Upon removing the calvarium, sinuses and veins of the pia mater, of superior part of hemispheres, were found highly congested, but no effusion of lymph or pus was detected. Base of the brain.—Surrounding the commissure of the optic nerves, and occupying the anterior subarachnoidean space, was an abscess containing about one ounce of fetid, scrofulous pus. The Dura Mater covering the upper surface of the petrous portion of the left os temporis at the entrance of the Vidian nerve, was of a dark red color for the extent of about two inches; the diseased portion of the membrane being of an elliptical shape, and terminated by a well defined margin of healthy structure. Beneath, and corresponding exactly in extent with this elliptical discoloration, was clustered together a number of scrofulous tubercles of the size of split peas; some of these bodies were crude, others in process of ulceration and softening, resting upon a base of carious bone. On their removal, the substance of the petrous pyramid was found extensively diseased; ulceration had attacked not only the Aqueductus Fallopii, and thereby destroyed the continuity of the Portio Dura, but had penetrated into the cavity of the tympanum. Substance of the 5th pair of nerves was found entire, but somewhat softened in texture, thus accounting most satisfactorily for the incomplete anæsthesia of the facial muscles.

